# Practicing governance towards equity in health systems: LMIC perspectives and experience

**DOI:** 10.1186/s12939-017-0665-0

**Published:** 2017-09-15

**Authors:** Lucy Gilson, Uta Lehmann, Helen Schneider

**Affiliations:** 1 School of Public Health and Family Medicine, University of Cape Town, Cape Town, South Africa; 2 Department of Global Health and Development, London School of Hygiene and Tropical Medicine, London, UK; 3 School of Public Health, University of the Western Cape, Cape Town, South Africa

**Keywords:** Governance, Health systems, Equity, Everyday practice

## Abstract

The unifying theme of the papers in this series is a concern for understanding the everyday practice of governance in low- and middle-income country (LMIC) health systems. Rather than seeing governance as a normative health system goal addressed through the architecture and design of accountability and regulatory frameworks, these papers provide insights into the real-world decision-making of health policy and system actors. Their multiple, routine decisions translate policy intentions into practice – and are filtered through relationships, underpinned by values and norms, influenced by organizational structures and resources, and embedded in historical and socio-political contexts. These decisions are also political acts – in that they influence who accesses benefits and whose voices are heard in decision-making, reinforcing or challenging existing institutional exclusion and power inequalities. In other words, the everyday practice of governance has direct impacts on health system equity.

The papers in the series address governance through diverse health policy and system issues, consider actors located at multiple levels of the system and draw on multi-disciplinary perspectives. They present detailed examination of experiences in a range of African and Indian settings, led by authors who live and work in these settings. The overall purpose of the papers in this series is thus to provide an empirical and embedded research perspective on governance and equity in health systems.

Governance has been widely acknowledged as one of the most important components of every health system [1, 2]. Current Universal Health Coverage (UHC) debates reaffirm its central role in improving health sector performance [3] and the global ‘Health Systems Governance Collaborative’, connected to the UHC 2030 platform (https://www.uhc2030.org), has now been established to harness and strengthen networks and communities of practice working on governance issues [4]. A very recent crop of papers has, meanwhile, reviewed and proposed a range of relevant frameworks [5–7]. Yet, nearly 10 years after being identified as a neglected area of research in low- and middle-income countries (LMICs) [8], there remains limited empirical evidence about health system governance [6].

Against this backdrop, this thematic series in the *International Journal for Equity in Health* (*IJEqH*) presents a set of papers that report empirical governance research undertaken in various Indian and African settings, and led by authors living and working in these settings. Most of these papers were discussed at a workshop held near Cape Town, South Africa in April 2016, and hosted by the Collaboration for Health Systems Analysis and Innovation (www.chesai.org). The first eight papers are published alongside this editorial, and considered within it. Additional papers will offer further insights on decentralization, accountability, managerial decision-making, and experiences of health policy implementation, as well as related research practice.

In broad terms, our work recognizes governance to be a dynamic and complex process, rather than a normative health system goal achieved through the architecture and design of accountability and regulatory frameworks. Figure 1 summarizes the processes and related phenomena that we examine; and from this foundation, the papers presented here contribute in four main and inter-linked ways to existing governance debates.

**Fig. 1:**
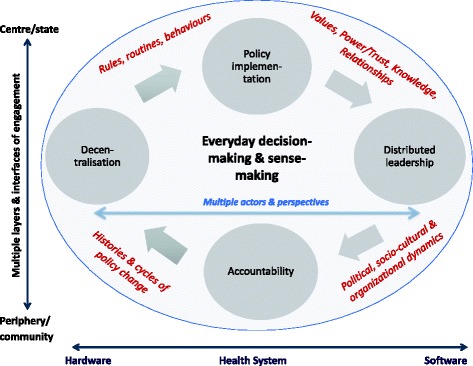
The terrain of the everyday practice of health system governance

First, the papers illuminate the micro-practices of governance, *the everyday practice of decision-making and meaning-making* undertaken by multiple health policy and system actors. Pyone et al. [6], similarly, argue that ‘Governance is a practice, dependent on arrangements set at political or national level, but which needs to be operationalized by individuals at lower levels in the health system’ (p.720). For example, while policy directives on postings and transfers in the Ghanaian health system are relatively clear, the practice of health workforce governance entails a complex web of decision-making occurring through “negotiation-spaces” at regional and district level [9].

This understanding of governance draws strongly from public policy theory and its ideas both about how street level bureaucrats’ (SLBs) decisions and routines become the public policies they implement, and the horizontal policy networks and communities that bring policy actors together across organisations and settings to learn and take action [10]. The notion of practice, more specifically, directs attention to understanding how purposeful and effective action is triggered by everyday situations, requiring improvisation guided by values and drawing on tacit knowledge, rather than being fully rule-bound or always goal-oriented. Such action is effected through people, relationships and meanings, and is influenced by, and influences, wider social configurations [11]. For example, Erasmus et al. [12] illuminate how policy implementation within two South African district hospitals is shaped by the daily decision-making of multiple actors, influenced by organizational trust and the degree of fit between the policy content and hospital culture. Leadership of policy implementation must then, they suggest, vary across organizational units and between policies, working with features of specific organizational settings.

Second, therefore, the papers address an acknowledged area of importance, and yet weakness, in health system governance debates – *implementing change in health systems* [4]. They provide insights into the significant governance ‘work’ involved – across levels of the health system – whether implementing, for example, decentralization reforms in Kenya [13, 14], community-based system strengthening in South Africa [15], or human resource management policies in Ghana [9].

Through their implementation focus, the papers also deepen understanding around three inter-related and recent shifts in governance thinking:

- from a linear and top-down conception of the health policy process, which starts with policy formulation then proceeds in sequence to implementation and evaluation, to more complex processes involving bottom-up and top-down interactions occurring iteratively [16, 17];
- from viewing governance, especially in decentralized or plural health systems, as a property of the national sphere, to recognizing its “multi-level” and “polycentric” nature [1, 18];
- from a state- to society-centred approach to governance, which more fully recognizes “the multiplicity of societal actors involved in governance” [16, 19].

Indeed, third, the papers clearly highlight the particular *governance roles of actors at the ‘lower’ levels of the health system* as well as *illuminating the institutional forces that influence their relationships and practices*. Actors considered include sub-national managers [9, 13–15], local, mid-level managers [20, 21], front line health workers [12], patients and community actors [13, 14, 22]. Brinkerhoff and Bossert [19] similarly understand health system governance as entailing a range of governance agents, including providers and patients/citizens as well as the state, and the formal and informal rules shaping the relationships among them. Abimbola et al. [5], meanwhile, talk specifically about ‘relational governance’. Governance relationships include those embedded in vertical, ‘bureaucratic’ forms of accountability, involving financial and performance compliance, as well as the ‘external’ or horizontal accountability mechanisms enabling cooperation across organizations and responsiveness to citizens – and that rely on dialogue, sharing of information, and cultures of trust and reciprocity [18, 23]. These forms of accountability may, however, conflict with each other. In South Africa, for example, Scott and Gilson [21] show how information use at primary care level is shaped by higher level planning needs and demands for formal information, although, in practice, disaggregated information and experiential knowledge is more useful in locally responsive decision-making. Mid-level district managers, therefore, play a critical role in mediating centrally-led action and creating spaces for locally-responsive decision-making (see also [9, 20]). Distributed leadership is, then, important within health system governance – that is, the leadership offered by multiple actors across levels of a health system and entailing the social process of influencing others through, for example, the careful use of power and sense-making [24]. Based on detailed analysis of South African experience, Schneider and Nxumalo [15], meanwhile, spell out key leadership and governance roles for sub-national stewards seeking to strengthen Community Health Worker programmes and community-based services, and emphasize the need to strengthen both vertical and horizontal accountability.

Fourth, and finally, the papers provide rich insights into, and deepen understanding of, the ways in which *health system hardware and software* [25] combine to influence governance practices and shape health system functioning [26]. In Kenya, Tsofa et al. [13, 14] show how the implementation of devolution to newly established Counties offered the opportunity of community involvement in health planning, and local level decision-making better able to address local needs. However, speedy devolution, resulting from political and societal pressures, generated weak planning and management processes that prevented community involvement and created disruptions in drug supplies and health worker motivation, at least immediately post-devolution. Some papers, meanwhile, specifically illuminate how ‘intangible software’ such as values, norms, communication practices and power or trust relationships, influence everyday governance practices and are themselves situated in wider political and organizational contexts and histories. In Kenya again, Nyikuri et al. [20] show how mid-level managers drew on the intangible software of personal values, commitment and relationships to maintain support for frontline health workers and service delivery within the wider context of devolution and disruption. Scott et al. [22], meanwhile, demonstrate how local gender and power dynamics limit the capacity of representative local bodies (Village Health Sanitation and Nutrition Committees) in Northern India to effect transformative social action.

But what do these insights into health system governance have to offer in thinking about health equity?

A recent editorial [27] in this journal called for new research in equity in health. Amongst the new areas of work highlighted are papers that recognize policymaking is not a technocractic process [28] and consider the inequities in power and institutional practices of social exclusion that underlie indigenous health inequalities [29]. This latter paper calls for deeper ‘understanding of the interrelationships that underlie public sector function as well as the “soft” systems of formal and informal rules, norms and values that guide stakeholder action’ ([29] p.3). Available evidence demonstrates, for example, that in addition to geographic and financial access barriers, marginalised and vulnerable groups commonly experience health care as demeaning and exclusionary and these combined barriers may result in impoverishment or underlie differential health outcomes across socio-economic population groups [30, 31].

Such health equity concerns are integrally linked to understanding governance as everyday decision-making practice. Indeed, drawing from its policy theory roots, the everyday practices of mid-level managers and front line health workers, or SLBs, are understood as political acts [32]. This is because ‘through their decisions they influence both citizens’ levels of access to public services or welfare benefits, as well as their experience of that access… SLBs are, quite simply, the daily reality of the state in most people’s experience and so their behaviours signal the value the state, society places on different people’ ([33] p.388). It has, therefore, been argued that in health systems, ‘successful implementation of policies to promote equity and inclusion requires a focus on human interactions at the micro-level as well as the development of supportive institutional systems for financing, information and regulation’ ([34] p.117).

The papers falling within this special edition offer insights into the everyday decision-making practices that maintain inequity [22] or resist equity-promoting policy implementation [9, 12–14], as well as those entailed in purposeful efforts to maintain decent health services [12, 21], even in the face of wider systemic stress [20], and in efforts to promote equity or strengthen accountability [13–15, 22]. Indeed, whilst health systems reflect existing patterns of social inequality, they also provide a site from which to contest them [35]. Understanding current everyday governance practices could, then, provide the basis for ideas about new practices that tackle health inequity – for example: ‘Development of a rights-based health system that increasingly addresses the systematic barriers to care experienced by poor and vulnerable groups requires managers who are more than administrators, managers who understand a given context and are able to take appropriate action’ ([34], p.117).

The final contribution of this set of papers is in illustrating how to go about the challenging task of doing research on health systems governance [8]. As these papers start with concern for the micro level (the individual policy actor) – often situated in meso-level systems – rather than the macro level (the overarching structure), they have broadly adopted a bottom-up, rather than top-down, approach to analysis [11]. Such analysis requires consideration of the lived reality of the decision-makers considered [36], in that governance practices are situated in, and shaped by, a broader set of institutional and social forces [5]. Research of this sort demands, as these papers attest, deep understanding of specific settings, commonly drawing on flexible research strategies and qualitative methodologies [37–39]. These include forms of participatory and action research [22], in some instances working over time in purposefully established health system learning sites [13, 14, 20, 21]. All paper authors are, moreover, embedded researchers [40] who have long-term research experience in the contexts where the research is situated, and are located in webs of relationships with local health system actors. As discussed in the April 2016 workshop, we acknowledge that our research practice, like the everyday practice of governance, is innately political – informed by specific worldviews and values. Reflexivity is essential for this work, alongside appropriate use of theory, and processes of data analysis and review that generate credible and trustworthy analyses [41, 42].

As a last comment, we thank the Editors of *IJEqH* for accepting this thematic series, although perhaps a little unusual for the journal – and in particular, Dr. Ana Lorena Ruano for her consistent support. We also thank the authors of these papers, and those to come, and all who participated in the stimulating and engaged April 2016 workshop.

## Abbreviations

IJEqH: International Journal for Equity in Health
LMIC: Low- and middle-income countries
SLB: Street level bureaucrat
UHC: Universal health coverage
